# Uterine natural killer cell-mediated immune imbalance impairs uterine vascular remodeling and contributes to adverse pregnancy outcomes in dengue virus-infected mice

**DOI:** 10.1128/spectrum.03429-25

**Published:** 2026-04-29

**Authors:** Feiyang Xue, Dongying Fan, Han Wang, Yilin Yang, Shiqi He, Hao Zhang, Zhengran Song, Yingying Zhang, Na Gao, Peigang Wang, Jing An, Ziyang Sheng

**Affiliations:** 1 Department of Microbiology, School of Basic Medical Sciences, Capital Medical University, Beijing, China; 2 Department of Blood Transfusion, Peking University Third Hospital, Beijing, China; 3 Department of Clinical Laboratory, Sir Run Run Shaw Hospital, Zhejiang University School of Medicine, Hangzhou, China; Agency for Science Technology and Research, Singapore, Singapore

**Keywords:** dengue virus, uterus, adverse pregnancy outcome, uNK cells

## Abstract

Recent studies have shown that dengue virus (DENV) infection during pregnancy is linked to adverse pregnancy outcomes, presenting an emerging clinical challenge with poorly understood pathogenic mechanisms. Uterine vascular remodeling, a critical determinant of successful pregnancy, is predominantly regulated by uterine natural killer (uNK) cells. In this study, we explored the effect of uNK cells on uterine injury in a DENV-induced adverse pregnancy mouse model. Transcriptomic analysis revealed significant dysregulation of uterine vascular remodeling and immune response-related gene expression after DENV-2 infection. Histopathological analysis revealed vascular remodeling disorders characterized by the preservation of vascular smooth muscle and a high level of tight junction protein in the vascular wall, accompanied by abnormal dilation of the lumen. These structural abnormalities coincided with a marked increase in uNK cell infiltration in the uterus. Moreover, the depletion of NK cells with anti-Asialo-GM1 mitigated uterine vascular damage and improved pregnancy outcomes. Single-cell RNA sequencing revealed a subpopulation of uNK cells (uNK2) that is characterized by upregulated expression of cytotoxicity-related genes. We further revealed that monocytes/macrophages (Mon_Macro) serve as intermediates in uNK2-mediated neutrophil (NE) recruitment. The SPP1–CD44 signaling axis is the dominant pathway governing uNK2-Mon_Macro crosstalk, and activated Mon_Macro2 can secrete chemokines such as CXCL2 to recruit NE. These findings provide valuable insights into the pathogenesis of DENV-induced uterine injury and facilitate the development of targeted therapeutic interventions for DENV-associated gestational disorders.

**IMPORTANCE:**

Dengue virus (DENV), a globally prevalent mosquito-borne virus, increasingly threatens maternal health because its infection is linked to adverse pregnancy outcomes. However, the mechanisms underlying DENV-induced damage to the uterus remain elusive. This study revealed impaired uterine vascular remodeling and abnormal vascular dilation following DENV-2 infection. Notably, uterine natural killer (uNK) cells are the critical pathological determinants of vascular injury in the uterus. Mechanistically, uNK cells may influence monocytes/macrophages (Mon_Macro) through SPP1–CD44 signaling to drive neutrophil infiltration, leading to uterine damage and adverse pregnancy outcomes. Successful depletion of NK cells reduces vascular damage and improves pregnancy outcomes. These findings provide critical mechanistic insights into DENV-2-induced uterine pathological lesions, fundamentally advancing our understanding of mosquito-borne virus-associated gestational diseases. The identified molecular cascades not only reveal the etiological landscape of pregnancy complications but also establish therapeutic measures for intervention strategies in mosquito-borne viral disorders.

## INTRODUCTION

Dengue virus (DENV), a member of the *Orthoflavivirus* genus within the *Flaviviridae* family, is among the most significant human pathogens transmitted by *Aedes* mosquitoes (1). According to the World Health Organization, approximately 40% of the world’s population (over 2.5 billion people) lives in regions at high risk for dengue (2). Globally, approximately 390 million DENV infections occur annually, with more than 96 million patients requiring hospitalization (3). Climate change and urbanization have facilitated the geographic expansion and increased the incidence of dengue epidemics (4), increasing the risk of mortality.

In recent years, increasing attention has been given to new DENV complications, specifically the association between maternal DENV infection and adverse pregnancy outcomes. Several clinical cohort studies have revealed a significant association between maternal DENV infection and severe pregnancy complications, including preterm birth, fetal growth restriction, and spontaneous miscarriage (5–14). Therefore, women of reproductive age are considered to be at high risk for DENV infection complications. However, current research on adverse pregnancy outcomes resulting from maternal DENV infection is largely limited to case reports and clinical characteristics, with insufficient focus on the exploration of potential mechanisms.

Pregnancy is a highly complex physiological process, and its success depends on the dynamic balance between the fetus and the mother. The causes of adverse pregnancy outcomes can be analyzed from both fetal and maternal perspectives: one focuses on placental injury and the other explores uterine changes. In our previous research, we found that DENV infection leads to insufficient placental blood perfusion and damage to the placental villi (15). However, the pathological changes in the uterus during DENV infection and their association with adverse pregnancy outcomes remain largely unexplored.

Normally, the uterine tissue structure critical for pregnancy comprises the endometrium and the myometrium. These layers are the primary sites for the profound vascular remodeling and immune interactions that support gestation (16–21). After embryo implantation, the endometrium transforms into the decidua, providing essential support for fetal development and growth (22). The maternal spiral arteries exhibit significant structural changes—vascular remodeling—in which highly muscular vessels transform into wide-lumen, thin-walled, and low-resistance vessels (16). This adaptive dynamic change is the basis of maintaining the placental blood supply to meet the demand for fetal nutrition and development. Abnormal vascular remodeling can lead to various pregnancy complications, such as preeclampsia and fetal growth restriction (23–25).

Uterine immune cells are widely believed to facilitate the establishment and maintenance of pregnancy. Compared with peripheral blood natural killer cells (pbNK cells), uterine natural killer cells (uNK cells), a specialized subgroup of NK cells residing in the uterus, exhibit significant differences in gene expression, phenotype, and function (26). Numerous studies have highlighted the close relationship between uNK cells and vascular remodeling through the secretion of factors such as vascular endothelial growth factor, placental growth factor, and matrix metalloproteinases (MMPs) (27, 28). Although the effects of uNK cells in normal pregnancy have been extensively studied, their pathological roles in disease states are complex and vary depending on the specific disease condition. Research has shown that the expression of receptors, cytotoxicity, cytokine secretions, and functions of uNK cells are closely associated with various pregnancy complications, including preeclampsia, recurrent miscarriage, and fetal growth restriction (29–33).

Owing to limitations in obtaining human clinical samples, research on human diseases using mouse models has become commonplace. Mouse and human reproductive structures differ, but their pregnancy processes are relatively similar. After embryo implantation in mice, the endometrium undergoes decidualization, promoting remodeling of the decidual spiral arteries to allow placental growth, with complete maternal blood supply achieved by E10.5 (34).

To this end, this study utilized type I interferon (IFN-I) receptor knockout (*Ifnar1*^−/−^) mice to establish a DENV-induced adverse pregnancy model. This model is widely used in flavivirus research, as the deficiency in type I interferon signaling permits viral replication and pathogenesis, which are otherwise restricted in immunocompetent wild-type mice (35, 36). We focus on the manifestations of uterine injury, with particular emphasis on the remodeling of blood vessels during DENV infection. Additionally, we examined the potential interactions among immune cells, with NK cells as the focal point, to investigate the underlying pathological mechanisms of uterine changes. This study provides important evidence for elucidating the pathogenesis of adverse pregnancy outcomes after DENV infection while enhancing our understanding of these mechanisms.

## RESULTS

### Uterine microenvironment homeostasis was disrupted during DENV-induced adverse pregnancy outcomes in pregnant *Ifnar1*^−/−^ mice

**Fig 1:**
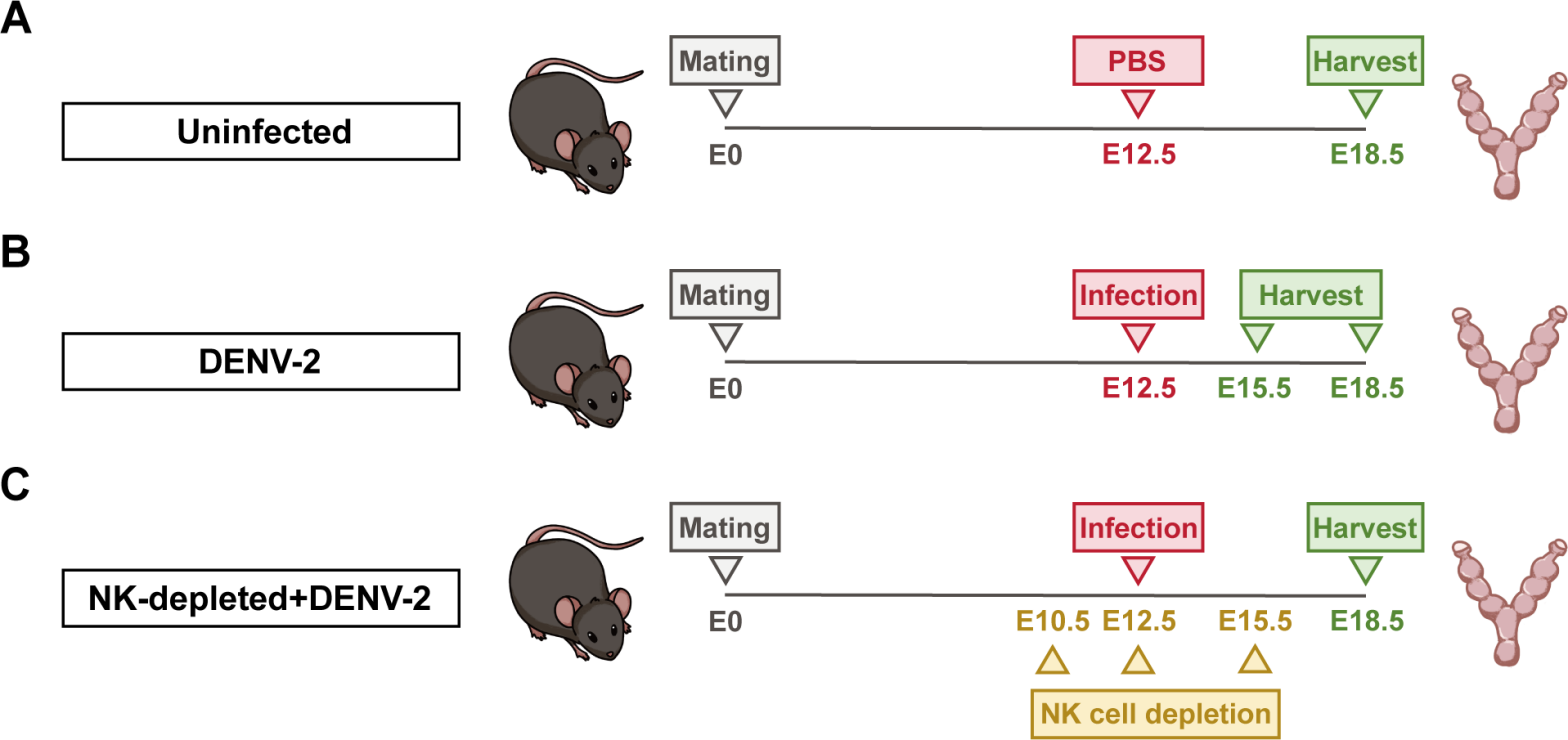
Details of model establishment. (**A**) Uninfected group. Pregnant C57BL/6 mice deficient in interferon α/β receptors (pregnant *Ifnar1*^−/−^ mice) were subcutaneously (s.c.) inoculated with 100 μL of sterile phosphate-buffered saline (PBS) at embryonic day 12.5 (E12.5) as the uninfected group. Uteruses and fetuses were collected at E18.5. (**B**) DENV-2-infected group. Pregnant *Ifnar1*^−/−^ mice were inoculated (s.c.) with 10^5^ plaque-forming units (PFU) of DENV-2 (100 μL) at E12.5 as the DENV-2-infected group. Uteruses and fetuses were collected at E15.5 and E18.5. (**C**) NK-depleted + DENV-2-treated group. Pregnant *Ifnar1*^−/−^ mice were intraperitoneally (i.p.) injected with 30 μg of Asialo ganglio-N-tetraosylceramide antibody (anti-Asialo-GM1) (100 μL) for three times at E10.5, E12.5, and E15.5 to deplete natural killer cell (NK cells). And mice were inoculated (s.c.) with 10^5^ PFU of DENV-2 (100 μL) at E12.5 as the NK-depleted + DENV-2-treated group. Peripheral blood, uteruses, and fetuses were collected at E18.5.

**Fig 2:**
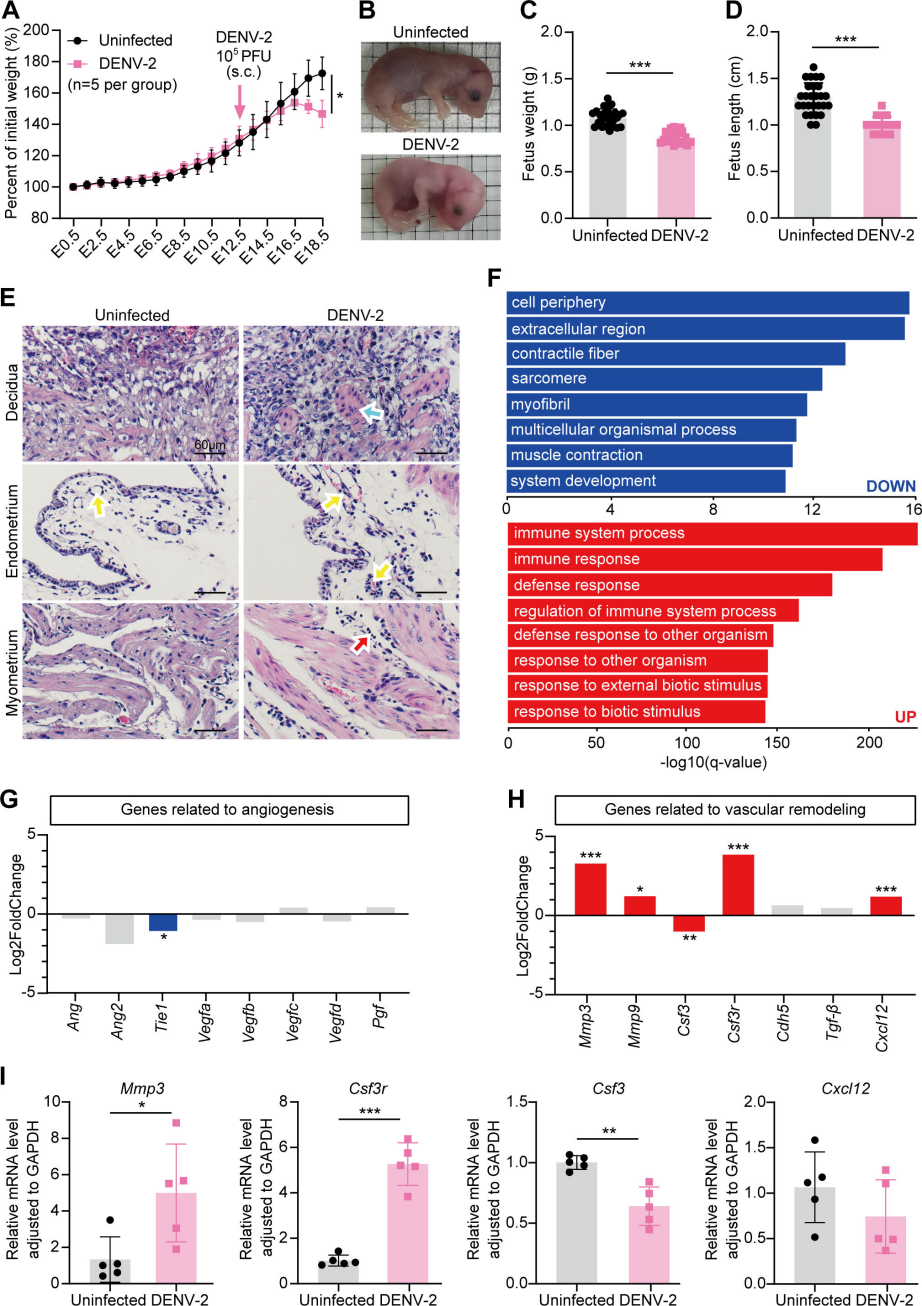
DENV-2 infection leads to abnormal uterine microenvironment homeostasis in pregnant *Ifnar1*^−/−^ mice. Pregnant *Ifnar1*^−/−^ mice were inoculated (s.c.) with 10^5^ PFU of DENV-2 (100 μL) at E12.5 as the DENV-2-infected group. Mice injected with the same volume of sterile PBS were used as the uninfected group. Uteruses and fetuses were collected at E18.5 for histopathological analysis and transcriptome sequencing (RNA-seq). (**A**) Maternal body weight changes in the uninfected group and the DENV-2-infected group were monitored from E0.5 to E18.5. *n* = 5 per group. (**B**) Gross morphology representative images of fetuses of E18.5 dams. Ischemia was observed in the DENV-2-infected group. (**C and D**) Fetal weight (**C**) and length (**D**) comparison of E18.5 dams. *n* = 23 in the uninfected group. *n* = 15 in the DENV-2-infected group. (**E**) Histopathological changes were analyzed by hematoxylin and eosin (HE) staining. The blue arrow indicates an increase in an abnormal structure that resembles fibrous connective tissue. The yellow arrow indicates the manifestation of vascular congestion. The red arrow indicates the infiltration of inflammatory cells. Scale bars: 60 μm. (**F**) Gene ontology (GO) enrichment analysis of differentially expressed genes (DEGs). The top eight pathways with significant changes between upregulated (red) and downregulated (blue) pathways are displayed. (**G and H**) Expression level of angiogenesis-related genes (**G**) and vascular remodeling-related genes (**H**) based on uterine transcriptomic data at E18.5. (**I**) Relative mRNA levels of *matrix metallopeptidase 3* (*Mmp3*), *colony stimulating factor 3 receptor* (*Csf3r*), *colony stimulating factor 3* (*Csf3*), and *C-X-C motif chemokine ligand 12* (*Cxcl12*) were determined by real-time quantitative polymerase chain reaction (RT-qPCR) in the uterus at E18.5. *n* = 5 per group. *P* value: *, < 0.05; **, < 0.01; ***, < 0.001. Data are presented as the mean ± standard deviation (SD) (**A, C, D, and I**). The significance of differences was analyzed by Mann-Whitney *U*-test (**A, C, D, and I**).

First, we established a DENV-2-induced late-stage adverse pregnancy model by subcutaneously (s.c.) injecting 10^5^ plaque-forming units (PFU) of DENV-2 into pregnant *Ifnar1*^−/−^ mice at embryonic day 12.5 (E12.5) through the footpad (Fig. 1A and B). Infected dams exhibited significant weight loss before delivery (E16.5–E18.5) (Fig. 2A), with corresponding fetal growth restriction evidenced by reduced weight and shorter length at E18.5 (Fig. 2B through D). These results confirm the successful establishment of the model. Hematoxylin and eosin (HE) staining revealed structural alterations across uterine layers post-infection, including a disorganized cellular architecture and increased fibroconnective-like tissue deposition in the decidua. Vascular congestion, characterized by luminal erythrocyte accumulation, suggesting compromised utero-placental circulation, was observed in the vessels of the endometrium. Inflammatory cell infiltration was evident in the myometrium (Fig. 2E).

To systematically characterize the uterine pathology induced by DENV-2, uteruses were harvested from *Ifnar1*^−/−^ dams in both the uninfected group and the DENV-2-infected group at E18.5 for transcriptome sequencing (RNA-seq). We identified a total of 2,052 differentially expressed genes (DEGs) between the two groups (|log2 fold change| > 1, padj < 0.05) in the uterus after DENV-2 infection, including 1,348 upregulated genes and 704 downregulated genes. Gene ontology (GO) analysis of the upregulated genes revealed enrichment of immune-related pathways, indicating that enhanced immune activation may contribute to microenvironment destabilization. The downregulated DEGs were predominantly enriched in extracellular matrix organization (GO: 0030198), myofibril assembly (GO: 0030239), and muscle contraction (GO: 0006936), indicating that relevant cellular components or biological processes in the uterus were suppressed after infection (Fig. 2F). In summary, DENV-2 infection disrupts the homeostasis of the uterine microenvironment in pregnant mice and impairs cellular functions.

The process of vascular formation during pregnancy involves two key components of the uterus: angiogenesis and vascular remodeling. The dynamic balance between these two processes is strictly regulated in terms of timing and spatial organization (37). To investigate the impact of microenvironmental disruption on this process, we observed that the expression of angiogenesis-related genes did not change significantly after infection (Fig. 2G). However, according to the uterine transcriptomic data, indicators related to vascular remodeling, such as *matrix metallopeptidase 3 (Mmp3*), *Mmp9*, and *colony stimulating factor 3 receptor (Csf3r*), were significantly upregulated (Fig. 2H). The real-time quantitative polymerase chain reaction (RT-qPCR) validation results revealed that the expression levels of *Mmp3*, *Csf3r*, and *colony stimulating factor 3 (Csf3*) matched the trends observed in the transcriptomic analysis, except for *C-X-C motif chemokine ligand 12 (Cxcl12)* (Fig. 2I). These findings suggest that the intrauterine developmental retardation caused by DENV-2 infection may be due to abnormal vascular remodeling in the uterus.

### Abnormal uterine vascular remodeling was observed after DENV-2 infection in pregnant *Ifnar1*^−/−^ mice

**Fig 3:**
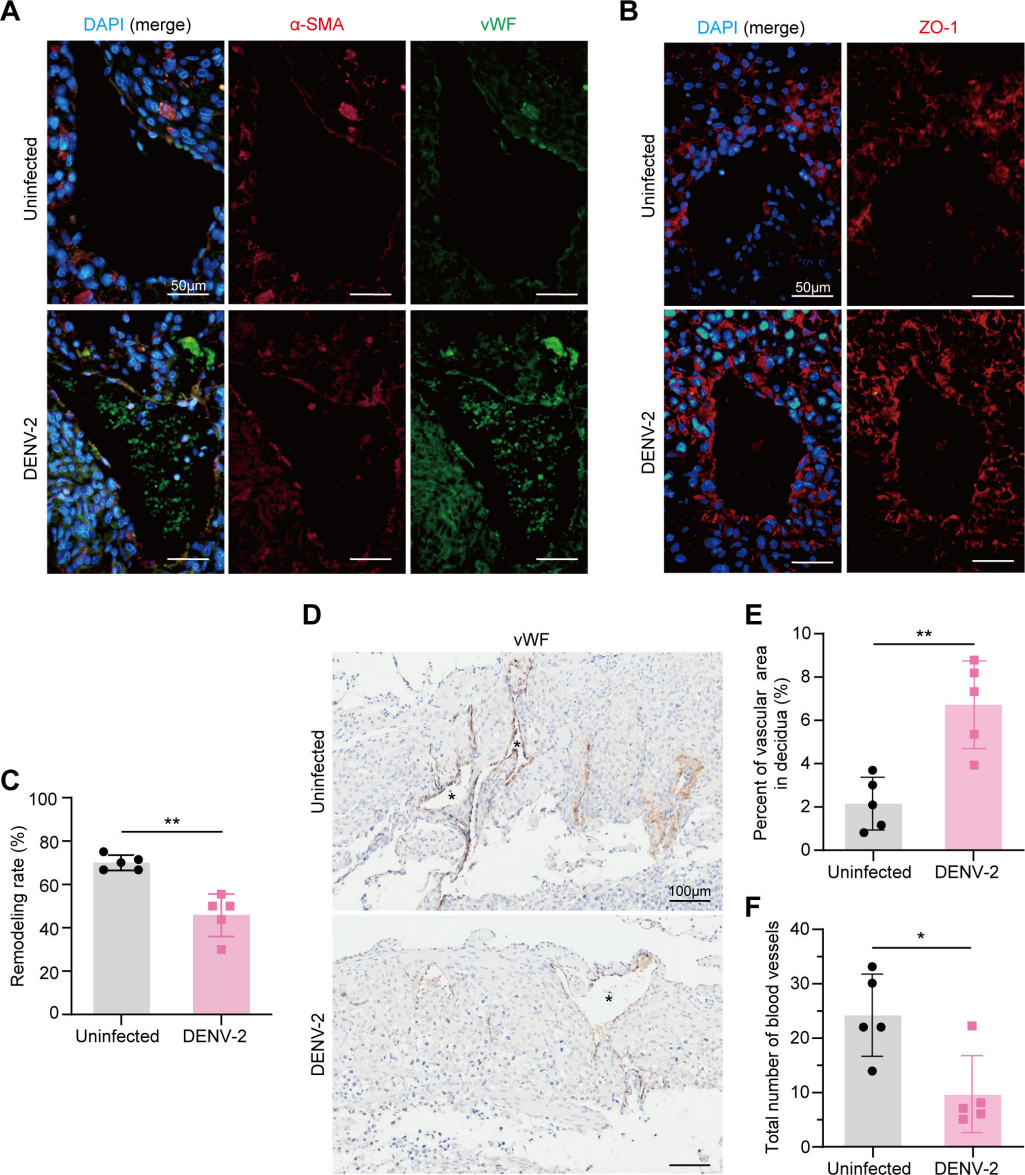
DENV-2 infection leads to abnormal uterine vascular remodeling in pregnant *Ifnar1*^−/−^ mice. (**A**) The smooth muscle cell replacement status was analyzed by immunofluorescence (IF) staining. Anti-alpha-smooth muscle actin antibody (anti-α-SMA) marks vascular smooth muscle cells (red), anti-von Willebrand factor antibody (anti-vWF) marks endothelial cells (green), and 4′,6-diamidino-2-phenylindole (DAPI) stains nuclei (blue). Scale bars: 50 μm. (**B**) The levels of tight junction proteins were analyzed by IF staining. Anti-ZO-1 antibody marks tight junction proteins (red), and DAPI stains nuclei (blue). Scale bars: 50 μm. (**C**) The completion rate of vascular remodeling. Blood vessels with complete loss of smooth muscle cells are considered to have finished remodeling. Remodeling rate = (number of remodeling-finished blood vessels / total number of blood vessels) × 100%. *n* = 5 per group. (**D**) Immunohistochemistry (IHC) staining of uterine blood vessels using anti-vWF antibody. The asterisk indicates the uterine decidual blood vessels. Specify that the specific marker is brown by IHC. Scale bars: 100 μm. (**E**) Quantification of vascular area. *n* = 5 per group. (**F**) Quantification of the number of vessels. *n* = 5 per group. *P* value: *, < 0.05; **, < 0.01. Data are presented as the mean ± SD (**C, E, and F**). The significance of differences was analyzed by Mann-Whitney *U*-test (**C, E, and F**).

Uterine vascular remodeling is a critical process during pregnancy and is characterized by the loss of vascular smooth muscle cells and widening gaps between vascular endothelial cells. To verify abnormal uterine vascular remodeling after DENV-2 infection, we used an anti-alpha smooth muscle actin (α-SMA) antibody to label smooth muscle cells and a von Willebrand factor (vWF) antibody to label vascular endothelial cells and then assessed the extent of vascular remodeling at E18.5. Compared with that in the uninfected group, the α-SMA staining of the vascular walls in the DENV-2-infected group remained positive (Fig. 3A), indicating that the smooth muscle cells had not completely regressed. Additionally, we observed abundant positive zonula occludens-1 (ZO-1) staining for tight junction proteins on the vascular walls (Fig. 3B), suggesting that the replacement of uterine vascular cells may be hindered. The slowed process of vascular remodeling following DENV-2 infection resulted in insufficient remodeling at E18.5, leading to a lower final completion rate of remodeling (Fig. 3C).

To further quantify the degree of vascular damage, we compared the vascular luminal area and the number of vessels between the two groups. Immunohistochemistry (IHC) staining revealed that the percentage of vascular area (within the decidua) in the infected group was abnormally increased (Fig. 3D and E), whereas the total number of vessels was lower (Fig. 3F). These results further indicate that DENV-2 infection affects the uterine blood vessel remodeling process in pregnant mice, leading to abnormal dilation and potentially impacting blood flow from the mother to the fetus.

### uNK cells act as key pathological drivers of adverse pregnancy outcomes caused by DENV-2 infection

**Fig 4:**
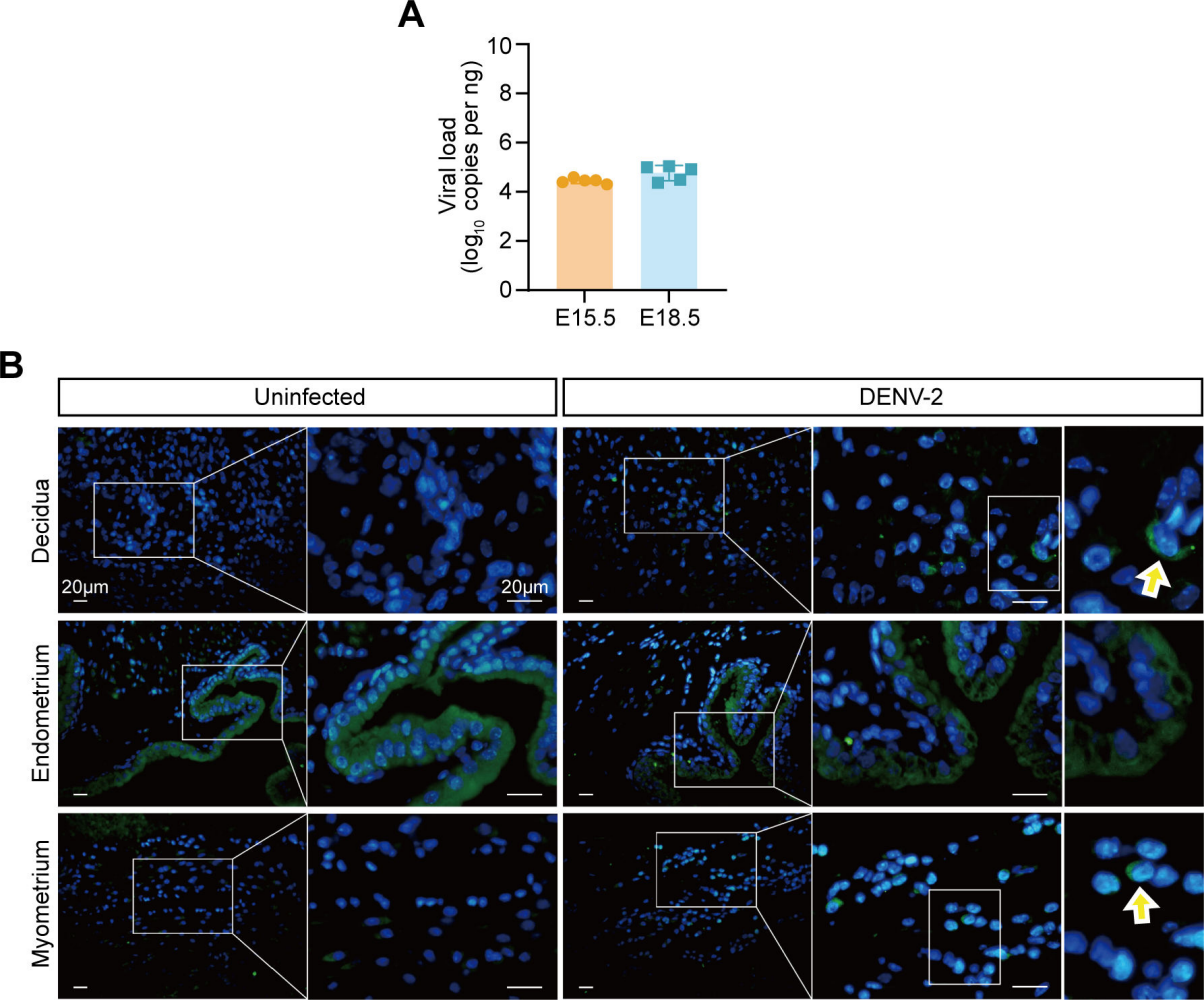
The viral burden in the maternal blood and uterus. (**A**) Quantification of DENV-2 viral loads in the uterus at E15.5 and E18.5, measured by RT-qPCR. (**B**) IF staining of DENV-2 antigen (green) in different uterine layers (decidua, endometrium, and myometrium) at E18.5. DAPI (blue) stains nuclei. Scale bars: 20 μm. Data are presented as the mean ± standard deviation (SD) (**A**). The significance of differences was analyzed by Mann-Whitney *U*-test (**A**).

To explore the specific mechanisms underlying uterine vascular damage after DENV-2 infection, we first evaluated the direct effects of viral replication. The RT‒qPCR results indicated that the viral load in uterine tissues at E15.5 and E18.5 remained stable and did not change with the duration of infection (Fig. 4A). Immunofluorescence (IF) staining revealed only a limited distribution of viral antigens in the decidua and myometrium after DENV-2 infection (Fig. 4B). These findings suggest that the uterus may not be a primary target organ for DENV-2 replication. In other words, adverse pregnancy outcomes and uterine vascular damage may not be directly caused by the destructive effects of viral proliferation. Instead, combined with the aforementioned transcriptomic results regarding the immune response and abnormal vascular remodeling, we speculate that the likely cause of adverse pregnancy outcomes and uterine vascular damage is immune-mediated pathological damage.

**Fig 5:**
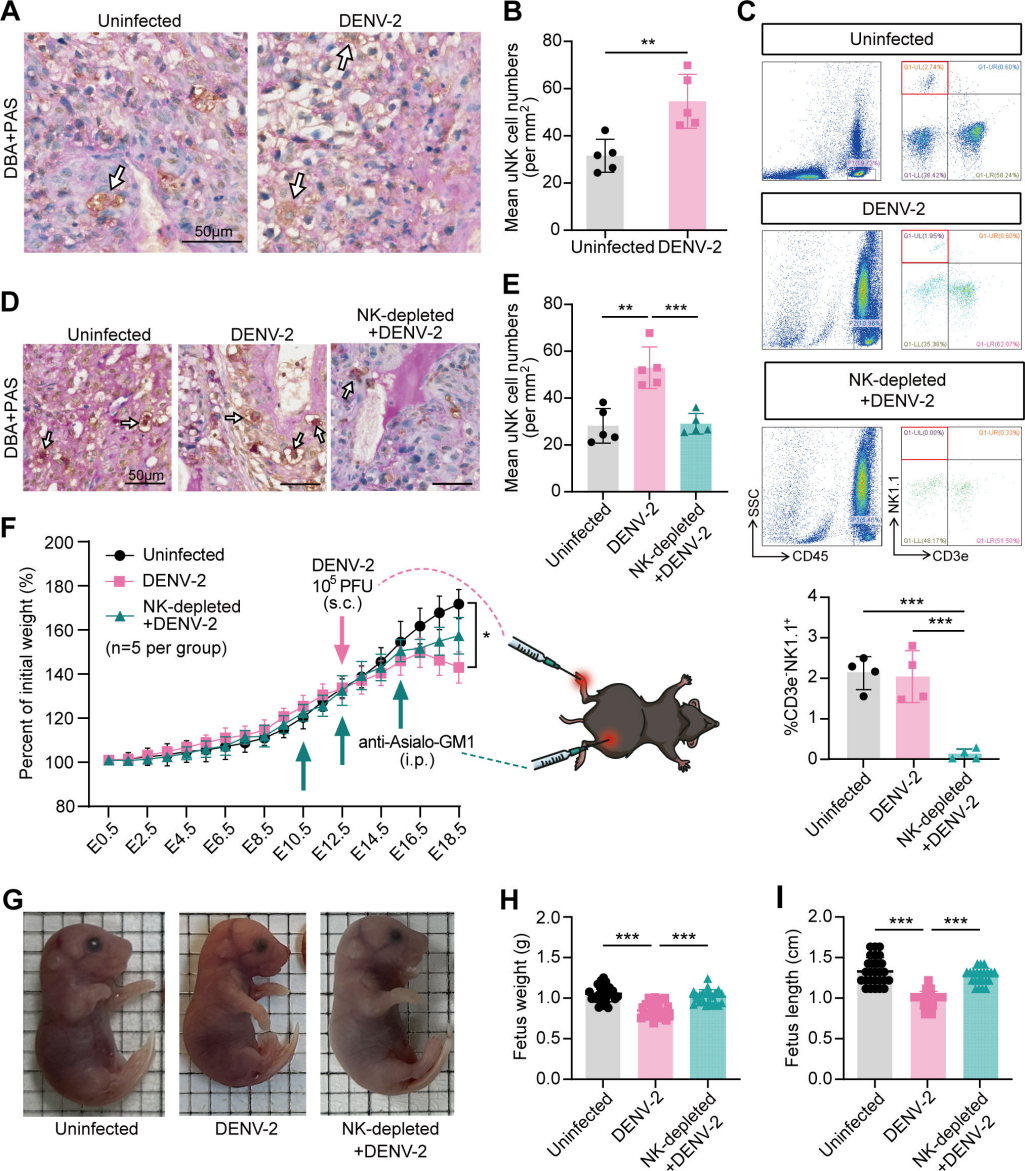
Depletion of uNK cells rescues fetal growth restriction after DENV-2 infection. To deplete NK cells, pregnant *Ifnar1*^−/−^ mice were injected (i.p.) with 30 μg of anti-Asialo-GM1 three times at E10.5, E12.5, and E15.5 as the NK-depleted + DENV-2-treated group. Mice were inoculated (s.c.) with 10^5^ PFU of DENV-2 at E12.5 as the DENV-2-infected group. Mice injected with the same volume of sterile PBS were used as the uninfected group. Peripheral blood, uteruses, and fetuses were collected at E18.5. (**A**) The location of uterine natural killer cells (uNK cells) was analyzed by *Dolichos biflorus* agglutinin (DBA) and Periodic Acid-Schiff (PAS) dual staining in the uninfected group and the DENV-2-infected group. White arrows indicate positive staining of uNK cells. Specify that the specific marker is brown by IHC. Scale bars: 50 μm. (**B**) Quantification of mean uNK cell numbers. *n* = 5 per group. (**C**) Depletion of NK cells in peripheral blood was confirmed by flow cytometry. The gating strategy is shown. CD45 antibody labels white blood cells. NK1.1 antibody labels NK cells. CD3e antibody was used to exclude T lymphocytes. *n* = 4 per group. (**D**) The location of uNK cells was analyzed by DBA and PAS dual staining in the uninfected group, the DENV-2-infected group, and the NK-depleted + DENV-2-treated group. White arrows indicate positive staining of uNK cells. Specify that the specific marker is brown by IHC. Scale bars: 50 μm. (**E**) Quantification of mean uNK cell numbers. *n* = 5 per group. (**F**) Maternal body weight changes in the uninfected group, the DENV-2-infected group, and the NK-depleted + DENV-2-treated group were monitored from E0.5 to E18.5. *n* = 5 per group. (**G**) Gross morphology representative images of fetuses of E18.5 dams. Fetal growth restriction and ischemia were alleviated in the NK-depleted + DENV-2-treated group. (**H and I**) Fetal weight (**H**) and length (**I**) comparison of E18.5 dams. *n* = 27 in the uninfected group. *n* = 27 in the DENV-2-infected group. *n* = 22 in the NK-depleted + DENV-2-treated group. *P* value: *, < 0.05; **, < 0.01; ***, < 0.001. Data are presented as the mean ± SD (**B, C, E, F, H, and I**). The significance of differences was analyzed by Mann-Whitney *U*-test (**B and F**). The significance of differences was analyzed by ordinary one-way ANOVA with Bonferroni’s multiple comparisons test (**C, E, H, and I**).

Among the various types of uterine immune cells, uNK cells are considered indispensable for vascular remodeling (38). By using *Dolichos biflorus* agglutinin (DBA) lectin and periodic acid-Schiff (PAS) dual staining, we detected uNK cells in the uterus at E18.5. In the DENV-2 infection group, the number of positive cells in the decidua was significantly greater than that in the uninfected group (Fig. 5A and B). With noticeable uterine vascular damage, we suggest an association between uNK cells and uterine vascular damage following DENV-2 infection.

To verify the above, we conducted depletion experiments on NK cells using Asialo ganglio-N-tetraosylceramide antibody (anti-Asialo-GM1). This drug completely depletes pbNK cells but does not fully deplete uNK cells. On the basis of the DENV-2-induced late-stage adverse pregnancy model, we established an NK-depleted + DENV-2-treated group in which the drug was administered intraperitoneally (i.p.) at a dose of 30 µg on E10.5, E12.5, and E15.5 (Fig. 1C). Flow cytometry confirmed the complete depletion of pbNK cells (Fig. 5C). Although the uNK cells were not completely depleted, their numbers were significantly lower than those in the DENV-2 infection group and were close to those in the uninfected group, as observed through IHC staining (Fig. 5D and E). This observation highlights the heterogeneity between uNK cells and pbNK cells. Additionally, the body weights of pregnant mice in the NK-depleted + DENV-2-treated group clearly improved (Fig. 5F). The growth restrictions of the fetuses were also significantly alleviated (Fig. 5G through I). These results indicate that reducing the number of uNK cells can effectively alleviate adverse pregnancy outcomes during late pregnancy following DENV-2 infection.

**Fig 6:**
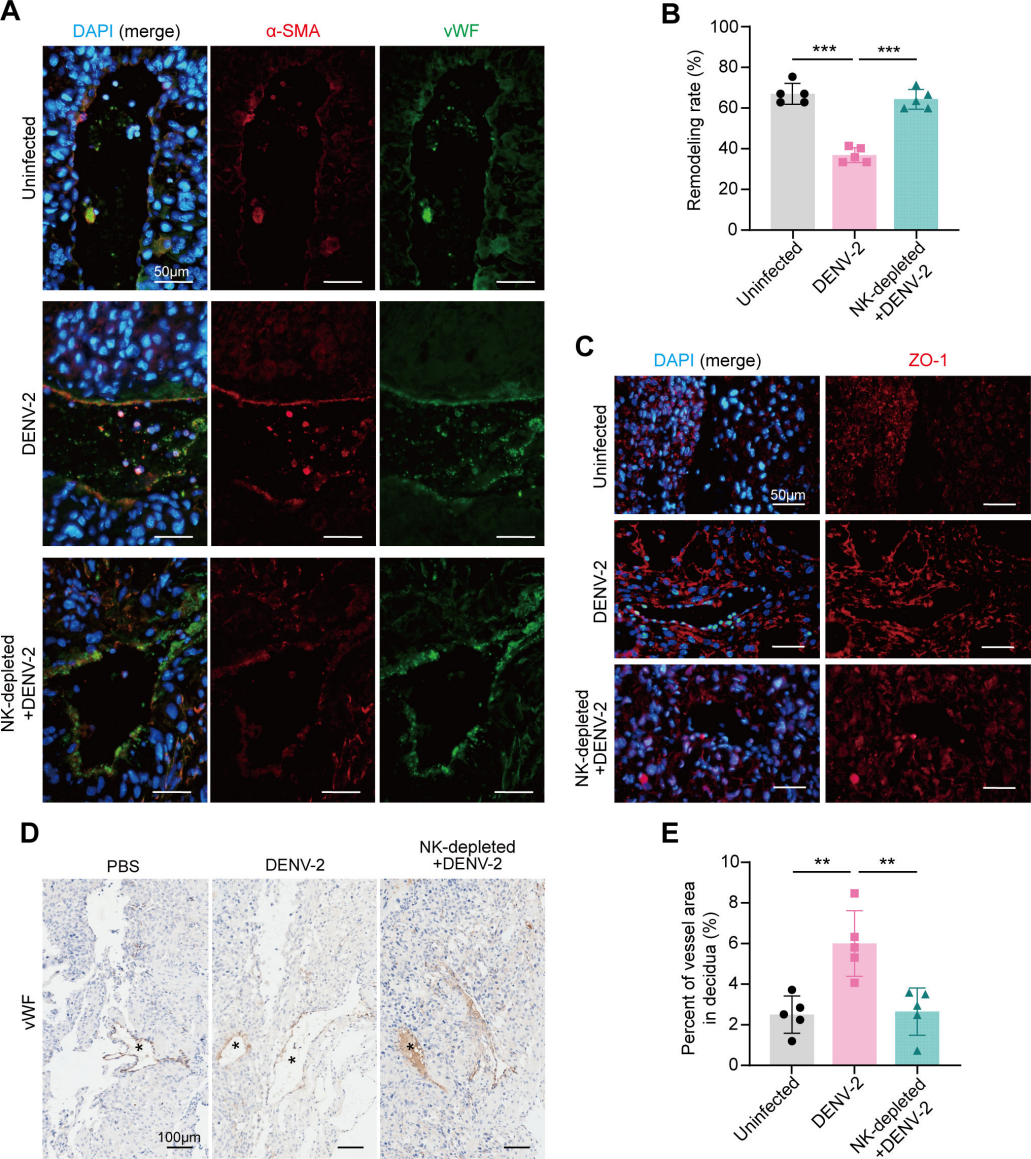
Depletion of NK cells alleviates uterine vascular damage in DENV-2-induced adverse pregnancy. (**A**) The smooth muscle cell replacement status was analyzed by IF staining. Anti-α-SMA antibody marks vascular smooth muscle cells (red), anti-vWF antibody marks endothelial cells (green), and DAPI stains nuclei (blue). Scale bars: 50 μm. (**B**) The completion rate of vascular remodeling. Blood vessels with complete loss of smooth muscle cells are considered to have remodeling finished. Remodeling rate = (number of remodeling-finished blood vessels / total number of blood vessels) × 100%. *n* = 5 per group. (**C**) The levels of tight junction proteins were analyzed by IF staining. Anti-ZO-1 antibody marks tight junction proteins (red), and DAPI stains nuclei (blue). Scale bars: 50 μm. (**D**) IHC staining of uterine blood vessels using anti-vWF antibody. The asterisk indicates the uterine decidual blood vessels. Specify that the specific marker is brown. Scale bars: 100 μm. (**E**) Quantification of vascular area. *n* = 5 per group. *P* value: **, < 0.01; ***, < 0.001. Data are presented as the mean ± SD (**B and E**). The significance of differences was analyzed by ordinary one-way ANOVA with Bonferroni’s multiple comparisons test (**B and E**).

We further assessed the degree of uterine vascular remodeling and damage at E18.5 after DENV-2 infection in the context of NK cell depletion. IHC staining revealed that smooth muscle retention in the uterine vessel walls was significantly improved in the NK-depleted + DENV-2-treated group compared to the infected group. The rate of vascular remodeling returned to normal levels (Fig. 6A and B). Additionally, the ZO-1 levels in the vessel walls decreased, indicating that the replacement of uterine vascular cells had recovered. Furthermore, compared with the DENV-2 infection group, the NK-depleted + DENV-2-treated group showed a significant reduction in the percentage of vascular area, which did not differ from that of the uninfected group (Fig. 6C and D). Therefore, it suggests that uterine vascular remodeling is nearly normalized following NK cell depletion, with vascular damage significantly reduced compared to the DENV-2-infected group.

These findings strongly support that uNK cells play a critical pathological role in adverse pregnancy outcomes following DENV infection.

### uNK subsets exhibit weakened vascular remodeling function after DENV-2 infection

**Fig 7:**
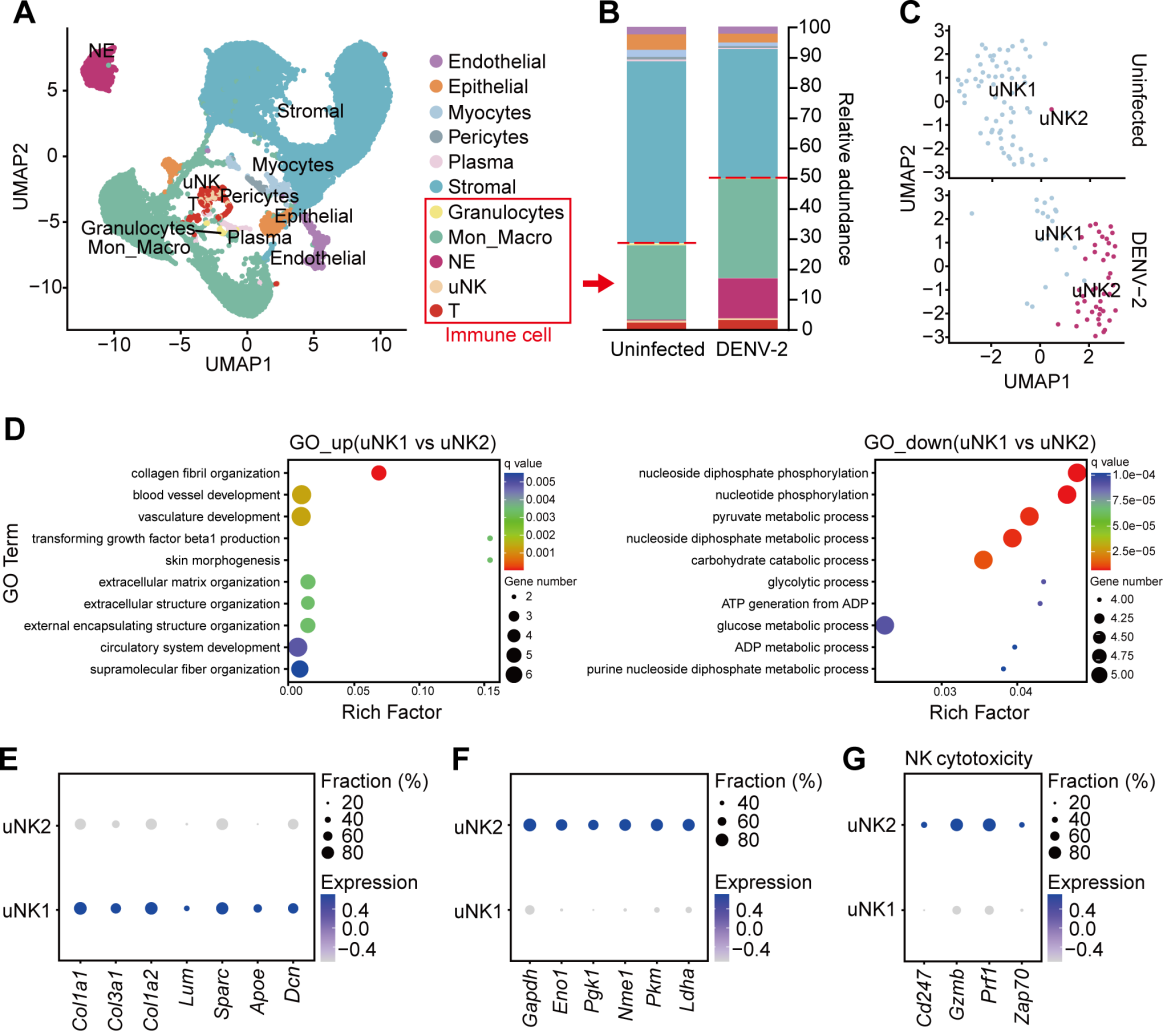
Single-cell sequencing analysis reveals phenotypic changes in uNK cells after DENV-2 infection. Pregnant *Ifnar1*^−/−^ mice were inoculated (s.c.) with 10^5^ PFU of DENV-2 at E12.5 as the DENV-2-infected group. Mice injected with the same volume of sterile PBS were used as the uninfected group. Uteruses were collected at E18.5 for single-cell RNA sequencing (scRNA-seq) analysis. (**A**) Uniform manifold approximation and projection (UMAP) plot displays the grouping of uterine cells. Each cluster represents a type of cell. Among them, immune cells include granulocytes, monocytes/macrophages (Mon_Macro), neutrophils (NE), and uNK cells. (**B**) Stacked bar plot depicts immune cell composition and proportion. (**C**) UMAP visualization of uNK cell subsets, identifying uNK1 and uNK2 subsets. (**D**) GO enrichment analysis of the uNK1 subset and the uNK2 subset. The top ten pathways with significant changes between upregulated and downregulated pathways are displayed. (**E and F**) Dot plot depicts genes with high expression in uNK1 cells (**E**) and uNK2 cells (**F**). (**G**) Dot plot depicts selected genes belonging to cytotoxicity.

**Fig 8:**
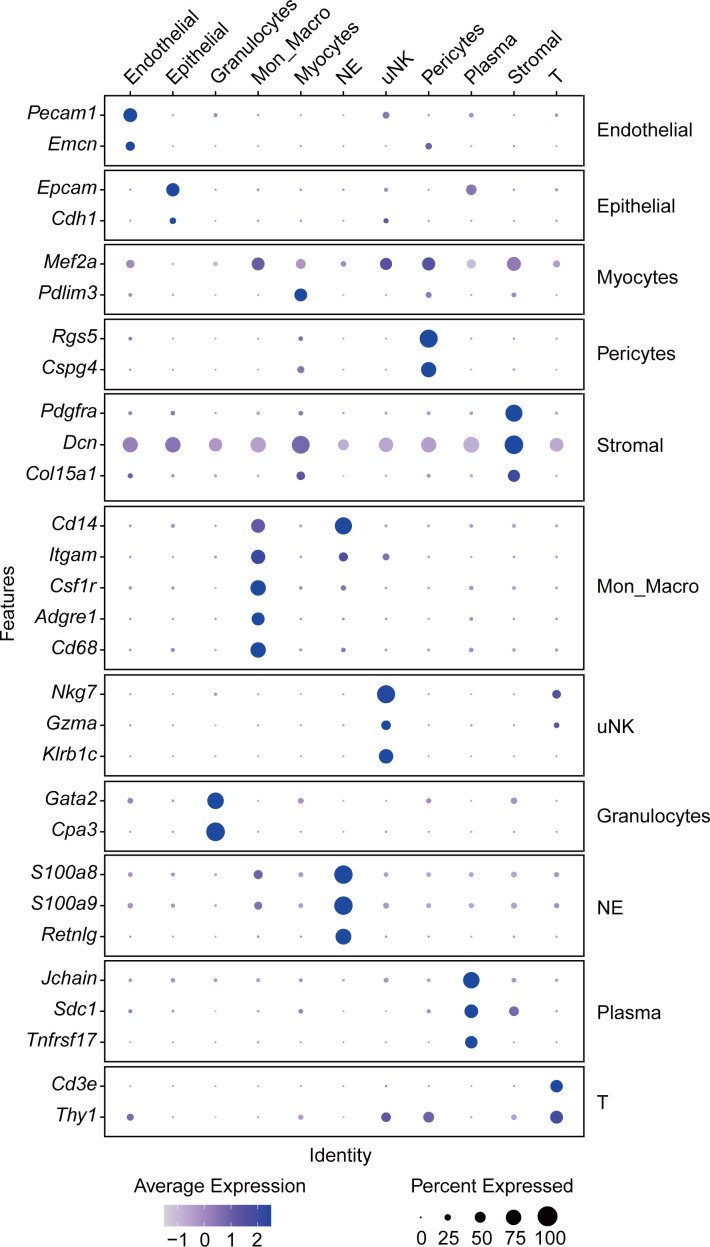
Cell grouping annotation marker.

To clarify the pathological mechanisms of uNK cells, we collected uteruses from pregnant *Ifnar1*^−/−^ mice at E18.5 to analyze the functional changes in uNK cells after DENV-2 infection using single-cell RNA sequencing (scRNA-seq). The specific details and clustering criteria are shown in Fig. 7A and 8. DENV-2 infection led to an increase in the proportion of immune cells in the uterus from 28% to 50% (Fig. 7B), which is consistent with previously observed changes in the uterine inflammatory microenvironment. Additionally, the composition of immune cells significantly changed, with a notable increase in the number of neutrophils (NE) (Fig. 7B).

Owing to the importance of uNK cells in pregnancy and the significant changes after DENV-2 infection, we extracted the uNK cell population from the scRNA-seq data and performed dimensionality reduction and clustering by PCA. The uNK cells in the uninfected group were composed mainly of the uNK1 subset, which accounted for more than 85% of the cells. In contrast, the uNK cells in the DENV-2 infection group differentiated into two distinct subsets: the uNK1 group and the uNK2 group (Fig. 7C). Further GO enrichment analysis revealed the functional differences between the subpopulations. The uNK1 subset was significantly enriched in pathways related to collagen fiber synthesis, angiogenesis, and extracellular matrix organization (Fig. 7D), which suggests that the uNK1 subset plays a core role in maintaining vascular remodeling and tissue homeostasis, aiding in the preservation of the original uterine decidual function. In contrast, the functional characteristics of uNK2 cells shifted toward pathways related to energy metabolism, including glycolysis and ATP production (Fig. 7D). Key pathways involved in vascular remodeling, such as extracellular matrix deposition and smooth muscle cell regulation, were significantly downregulated in the uNK2 subset (Fig. 7E). These findings indicate that the ability of uNK2 cells to support vascular remodeling may have been lost. The differential expression of metabolism-related genes in uNK2 cells supports this functional shift (Fig. 7F). The differentiation of uNK cell subpopulations suggests that after DENV-2 infection, uNK cells exhibit heterogeneity and contribute to the uterine pathological processes.

Notably, genes related to cytotoxic effector molecules (such as *Gzmb* and *Prf1*) and cell activation signaling molecules (such as *Cd247* and *Zap70*) were significantly expressed in uNK2 cells (Fig. 7G). The expression profile was highly similar to that of pbNK cells, suggesting that uNK2 cells may be involved in pathological immune damage. In contrast, the expression levels of these genes were very low in the original uNK1 subset (log2 fold change < 0.5), indicating that they maintain the low-cytotoxicity phenotype necessary for pregnancy.

On the basis of these results, we conclude that under normal conditions, uNK1 cells support a healthy pregnancy by maintaining angiogenesis and tissue repair functions. However, DENV-2 infection causes functional polarization within the uNK subpopulations. The uNK2 subset exhibits metabolic reprogramming and abnormal cytotoxic activation, which are closely related to uterine damage in DENV-2-infected dams.

### uNK2 cells recruit neutrophils through monocytes/macrophages in the uterus after DENV-2 infection

**Fig 9:**
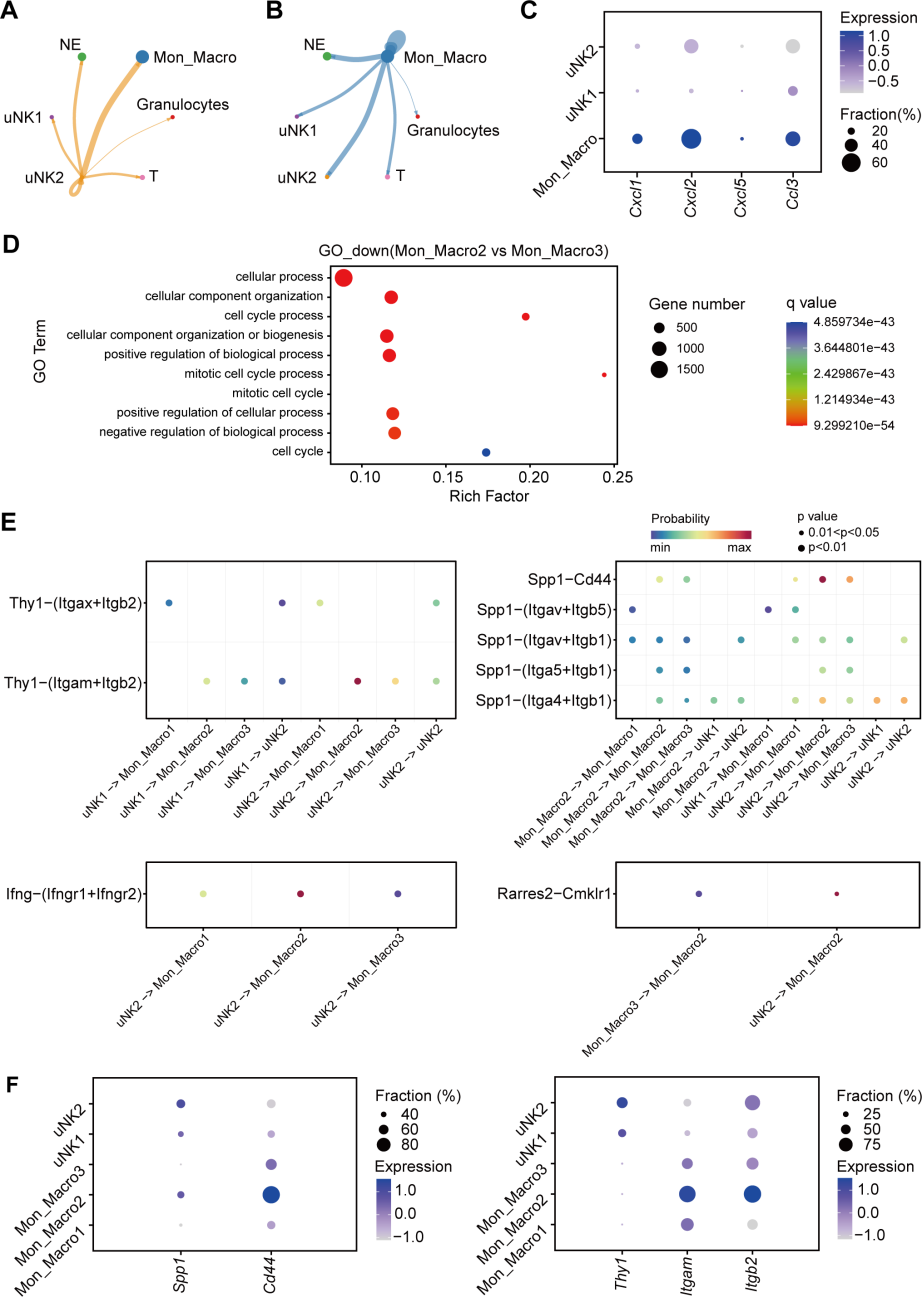
The signaling pathway from the uNK2 subset to the Mon_Macro2 subset. (**A and B**) CellChat analysis reveals that the signal intensity from uNK2 cells (**A**) or Mon_Macro (**B**) to other immune cells. (**C**) Dot plot depicts genes of specific chemokine ligands in uNK1 cells, uNK2 cells, and Mon_Macro. (**D**) GO enrichment analysis of the Mon_Macro2 subset and the Mon_Macro3 subset. (**E**) Activation of intercellular interaction signals in different ligand‒receptor pathways between uNK cells and Mon_Macro. (**F**) Dot plot depicts genes of the ligand and receptor expressed in SPP1‒CD44 and THY1‒(ITGAM+ITGB2) pathways.

Despite the low tissue abundance of uNK2 cells, we observed substantial local tissue damage. These paradoxical findings suggest that following DENV-2 infection, uNK2 cells may amplify pathological effects through cascade interactions with other immune cells. To investigate the pathological mechanisms of the uNK2 subset, we first studied immune cell communication within the uterine microenvironment under immune dysregulation. CellChat analysis revealed two key interactions. uNK2 cells exhibited robust communication with both monocytes/macrophages (Mon_Macro) and NE, with significantly stronger signaling toward Mon_Macro (Fig. 9A). In addition, Mon_Macro concurrently maintained intensive crosstalk with NE (Fig. 9B). These cascade reactions likely disrupt uterine immune homeostasis, ultimately contributing to microenvironmental imbalance and tissue injury.

**Fig 10:**
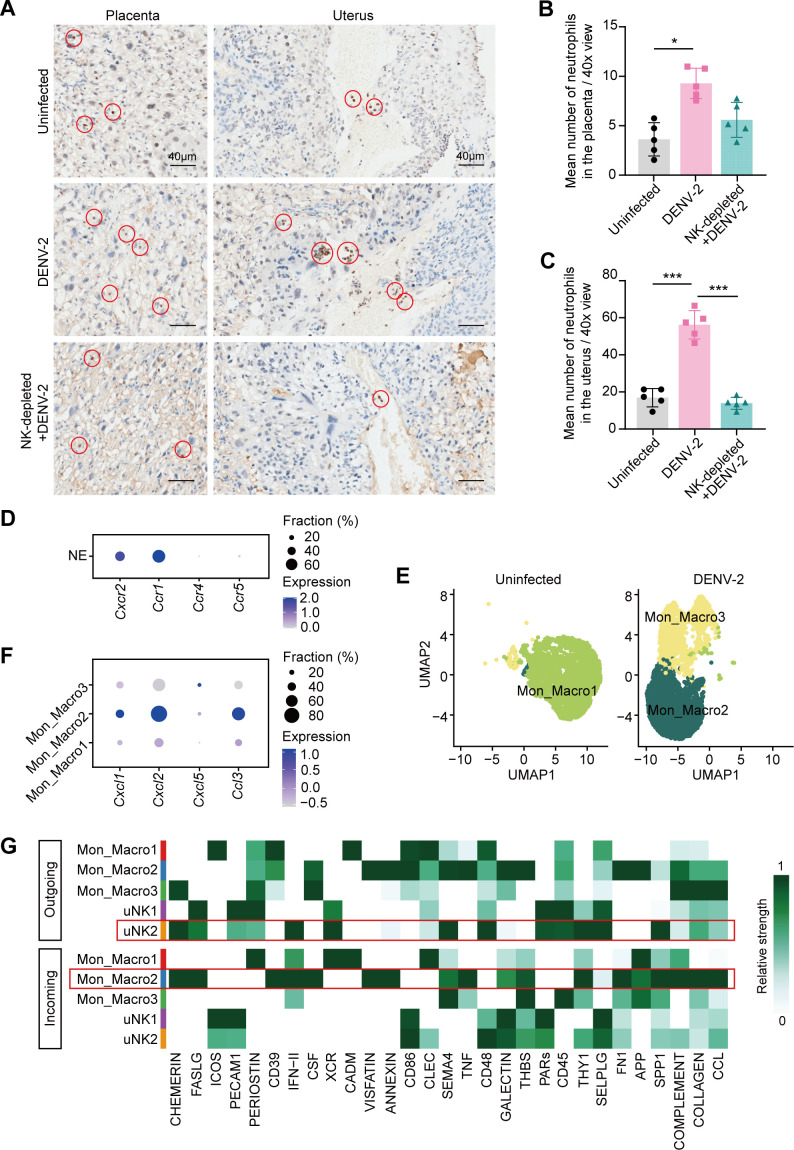
Signaling pathways involved in the neutrophil recruitment by uNK cells. (**A**) IHC staining of NE in the placenta and uterus using anti-lymphocyte antigen 6 complex locus G6D antibody (anti-Ly6G). The asterisk indicates the uterine decidual blood vessels. Specify that the specific marker is brown. Scale bars: 40 μm. (**B and C**) Quantification of neutrophil numbers in the placenta (**B**) and uterus (**C**). *n* = 5 per group. (**D**) Dot plot depicts the expression of neutrophil chemokine receptors. (**E**) UMAP visualization of Mon_Macro subsets, identifying Mon_Macro1, Mon_Macro2, and Mon_Macro3 subsets. (**F**) Dot plot depicts the expression of chemokine ligands in Mon_Macro subsets. (**G**) Heatmap shows the regulatory signaling pathways from uNK2 cells to Mon_Macro2. *P* value: *, < 0.05; ***< 0.001. Data are presented as the mean ± SD (**B and C**). The significance of differences was analyzed by ordinary one-way ANOVA with Bonferroni’s multiple comparisons test (**B and C**).

Neutrophils, as potent immune effector cells, mediate tissue damage through cytokine secretion and enzymatic activity. Following DENV-2 infection, both scRNA-seq data (Fig. 7B) and IHC staining (Fig. 10A) revealed abnormally elevated NE infiltration in the uterus. In the NK-depleted + DENV-2-treated group, NE accumulation decreased proportionally with a reduction in the number of uNK cells (Fig. 10B and C), which was accompanied by significant improvements in fetal growth parameters (Fig. 5G through I). These coordinated findings support the hypothesis that uNK cells contribute to DENV-2-induced uterine injury and adverse pregnancy outcomes through the orchestration of NE recruitment and activation.

To validate this hypothesis, we analyzed the potential signaling mechanisms at the molecular level. Neutrophil migration typically depends on the binding of chemokines to specific surface receptors. Our analysis revealed possible chemokine signaling patterns: NE exhibited robust expression of *C-C motif chemokine receptor 1* (*Ccr1*) and *C-X-C motif chemokine receptor 2 (Cxcr2*) receptors (Fig. 10D), whereas their cognate ligands *C-X-C motif chemokine ligand (Cxcl2*), *C-C motif chemokine ligand 3 (Ccl3*), and *Cxcl1* were predominantly expressed in Mon_Macro (Fig. 9C). This ligand‒receptor pairing indicates that Mon_Macro is likely to direct NE recruitment in uterine damage.

Interestingly, Mon_Macro exhibits minimal NE recruitment under physiological conditions, but DENV-2 infection specifically enhances this capacity, likely through phenotypic reprogramming. Mon_Macro was categorized into three distinct subsets after DENV-2 infection (Fig. 10E). The tissue-resident Mon_Macro1 subset displayed minimal involvement in NE recruitment. In contrast, Mon_Macro2 emerged as the primary source of NE-attracting chemokines (Fig. 10F). Mon_Macro3 upregulated the expression of cell cycle genes (Fig. 9D), suggesting that it has proliferative activity, although its functional role remains undefined. This functional differentiation supports the previously suspected mechanistic cascade wherein uNK2 cells activate Mon_Macro2, initiating pathological NE recruitment in the uterus after DENV-2 infection.

To investigate the mechanism underlying the interaction between uNK2 cells and Mon_Macro2, we identified key regulatory signals from uNK2 cells targeting Mon_Macro2. Our analysis revealed four highly activated signaling pathways in DENV-2 infection: SPP1, THY1, IFN-II, and CHEMERIN (Fig. 10G). Subsequent CellChat modeling of ligand‒receptor pair activation (Fig. 9E) demonstrated that DENV-2-infected uNK cells primarily communicate with Mon_Macro through two specific pathways: SPP1‒CD44 and THY1‒(ITGAM+ITGB2).

Secreted phosphoprotein 1 (SPP1) has stronger pregnancy-related relevance than thy-1 cell surface antigen (THY1) does, since SPP1 is highly expressed in the endometrium, with uNK cells serving as its primary source during uterine decidualization (39, 40). Our data demonstrate that uNK cells maintain high *Spp1* expression, while Mon_Macro2 abundantly express *Cd44* receptors (Fig. 9F). These findings collectively suggest that uNK2 cells likely activate Mon_Macro2 through SPP1‒CD44 signaling in DENV-2-infected uteruses, establishing a pathological communication axis.

### SPP1–CD44 signaling is among the potential pathways through which uNK2 cells exert their effects

**Fig 11:**
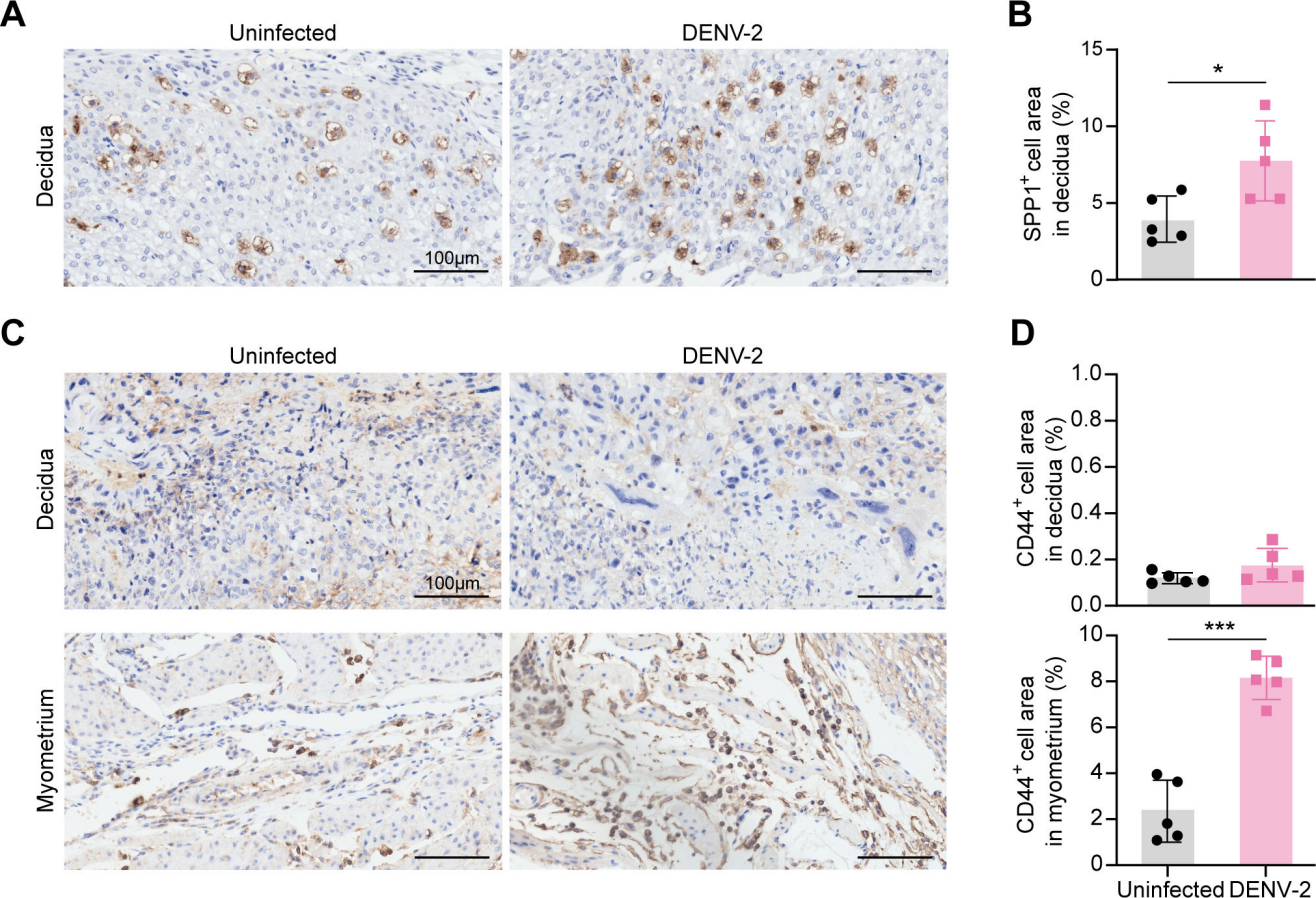
Activation of SPP1 and CD44 in the uterus of pregnant *Ifnar1*^−/−^ mice after DENV-2 infection. (**A**) IHC staining of secreted phosphoprotein 1 (SPP1) in the uterine decidua using anti-Osteopontin antibody. Specify that the specific marker is brown. Scale bars: 100 μm. (**B**) Quantification of SPP1^+^ cell area in the uterine decidua. *n* = 5 per group. (**C**) IHC staining of CD44 in the uterine decidua and myometrium using anti-CD44 antibody. Specify that the specific marker is brown. Scale bars: 100 μm. (**D**) Quantification of CD44^+^ cell area in the uterine decidua and myometrium. *n* = 5 per group. *P* value: *, < 0.05; ***, < 0.001; N.D., non-detected. Data are presented as the mean ± SD (**B and D**). The significance of differences was analyzed by ordinary one-way ANOVA with Bonferroni’s multiple comparisons test (**B and D**).

IHC staining was performed to validate the SPP1–CD44 signaling pathway at the histological level. After DENV-2 infection, Spp1^+^ cells localized specifically to the uterine decidua, with an increased positive staining area. These Spp1^+^ cells exhibited characteristic morphology and distribution patterns that matched those of uNK cells, which we examined by dual staining with DBA and PAS (Fig. 11A and B), confirming that uNK cells are the primary SPP1 producers whose expression is enhanced during infection. Moreover, the low expression of CD44 on Mon_Macro in the uninfected uterus significantly increased following DENV-2 infection (Fig. 11C and D).

This coordinated SPP1–CD44 activation confirmed that DENV-2 infection-induced expansion of uNK2 cells triggers SPP1 signaling, which stimulates Mon_Macro2 to secrete NE-attracting chemokines such as CXCL2. Subsequent NE recruitment to the uterus drives tissue damage, ultimately contributing to adverse pregnancy outcomes.

## DISCUSSION

Adverse pregnancy outcomes caused by DENV infection have become a global public health concern and primarily include preterm birth, fetal growth restriction, and fetal intrauterine demise. However, the underlying pathogenic mechanisms remain unclear, and effective countermeasures are lacking.

During pregnancy, the contact surface between the uterus and the placenta forms the maternal–fetal interface. Maternal blood delivers nutrients to the placenta through uterine arteries into the intervillous space, where material exchange occurs with fetal blood to support fetal development (31). In this study, type I interferon receptor knockout (*Ifnar1*^−/−^) mice were used to establish a DENV-induced adverse pregnancy model, with a focus mainly on the crucial pathological role of the uterus in adverse pregnancies caused by DENV-2 infection. Transcriptome analysis revealed an imbalance in gene expression related to uterine vascular remodeling, suggesting that abnormal vascular remodeling may be the main pathological change. We further observed significant uterine vascular remodeling disorders in pathological sections, manifested as incomplete replacement of vascular smooth muscle and high expression of ZO-1 at E18.5. Normally, the loss of smooth muscle cells causes the vascular wall to become more relaxed, while reducing resistance helps the vessels adapt to the rapidly increasing blood flow during pregnancy (16). The decrease in the strength of connections between endothelial cells leads to an increase in intercellular space, which helps improve the efficiency of cell replacement (41, 42). This vascular remodeling phenomenon is a physiological adaptation required for pregnancy. However, after DENV-2 infection, the replacement of uterine vascular cells in pregnant mice is affected, and the vessels are unable to form low-resistance, high-perfusion structures, as expected, ultimately affecting blood flow from the uterus to the placenta.

Previous reports support our results. Windsperger et al. reported a significant increase in vascular density in the decidua of recurrent miscarriage patients, while the proportion of remodeling vessels markedly decreased (43). Similarly, preeclampsia, placental abruption, fetal growth restriction, and miscarriage are strongly associated with defects in remodeling (24, 25, 44, 45), indicating that the uterine vasculature undergoes a unique remodeling process, and that insufficient remodeling can lead to serious pregnancy complications. Therefore, dysregulation of uterine vascular remodeling may be an important cause of DENV-2-induced adverse pregnancy.

Ample evidence suggests that uNK cells are required for facilitating vascular remodeling, regulating other uterine immune cells, and preventing pathogen infections (46–51). uNK cells represent a unique immune cell population in the uterus. The expression profile of uNK cells differs from that of pbNK cells, as these cells have a strong ability to secrete cytokines and weaker cytotoxicity (52). Our study revealed that, compared with uninfected pregnant mice, DENV-2 infection caused a significant increase in the number of uNK cells at E18.5. In fact, the number of uNK cells remains high during early pregnancy, lasting throughout the rapid development stage of the placenta, and then gradually decreases until the fetus is full term (38). Certain microbial infections associated with the maternal–fetal interface, such as human cytomegalovirus (HCMV), human immunodeficiency virus (HIV), Zika virus, and *Toxoplasma gondii*, have also been linked to adverse pregnancy outcomes through effects on uNK cells (53–55). Upon HCMV infection, the cytolytic ability of uNK cells is enhanced through the upregulation of the expression of cytotoxic receptors. In contrast, uNK cells secrete cytokines to combat viral invasion during HIV-1 infection. In this study, DENV-2 infection significantly increased the total number of uNK cells at E18.5.

We further analyzed the phenotypic changes in uNK cells after DENV infection. uNK1 cells highly express genes associated with vascular remodeling and tissue support, whereas uNK2 cells are characterized by vigorous metabolism and a cytotoxic phenotype; these findings prompted us to reexamine the functional phenotype diversity of uNK cells during DENV infection. In late pregnancy, although the number of uNK cells is reduced, depleting NK cells in pregnant mice with anti-Asialo-GM1 was able to significantly alleviate adverse pregnancy outcomes for both mothers and fetuses. These findings demonstrate that uNK cells, particularly the altered uNK2 subset, are a nonnegligible factor in uterine damage following DENV-2 infection.

It is worth mentioning that the anti-Asialo-GM1 antibody, while a commonly used tool for NK cell depletion in mice models, may have off-target effects on other cell types, such as basophils (56). However, basophils are a rare leukocyte subset accounting for less than 1% of peripheral blood leukocytes and are primarily implicated in mediating type 2 immune responses (e.g., anti-parasitic immunity and allergic reactions)(57–60). In contrast, our DENV-infected mouse model is characterized by a predominantly type 1 pro-inflammatory immune response (61), where basophils are not the major effector cells and exert only minimal functional contributions to the immune pathogenesis in acute DENV infection. Collectively, these characteristics suggest that the potential off-target effects of NK1.1 antibody on basophils would have negligible impacts on our assessment of the functional role of NK cells in immune recruitment during DENV infection.

Additionally, while studies indicate that anti-Asialo-GM1 can deplete certain monocyte/macrophage populations in the spleen (62), direct evidence for its efficacy against uterine-resident monocytes or macrophages remains insufficient. Nevertheless, despite these potential off-target effects, anti-Asialo-GM1 remains a mainstream method for NK cell depletion. This is primarily because the anti-NK1.1 has strain restrictions, whereas anti-Asialo-GM1 enables NK cell depletion across diverse mouse strains (63–65). Therefore, the applications of anti-Asialo-GM1 are more extensive and enhance experimental flexibility.

Continued exploration revealed that the effects of uNK2 cells may be amplified through a cascade response, initially regulating Mon_Macro, which in turn influences the recruitment of NE. We subsequently examined the specific signaling pathways related to this hypothesis. The SPP1–CD44 signaling pathway has significant physiological and pathological effects, particularly in tumor microenvironments, fibrosis, and immune regulation (66–69). Herington et al. demonstrated that uNK cells express the SPP1 protein and are involved in embryo implantation and placentation in pregnant mice (39). We further validated the significant activation of this pathway in DENV-2 infection, which revealed that uNK cells can regulate Mon_Macro through the SPP1–CD44 pathway. Moreover, the relationship between Mon_Macro and NE may be mediated by chemokines. Some evidence suggests that activation of Mon_Macro leads to the secretion of chemokines. CXCL2 secreted by monocytes and macrophages has a chemotactic effect on NE (70). Macrophages indirectly promote the survival and function of NE through the secretion of interleukin-1β (IL-1β) and matrix metalloproteinases (MMPs), which can enhance the phagocytic ability and antibacterial activity of NE. As a result, we conclude that uNK2 cells can affect the secretion of chemokines, such as CCL3, CXCL2, and CXCL1, by Mon_Macro2 through SPP1–CD44 to recruit NE to the uterus. These results also provide a mechanistic explanation for the abnormal infiltration of NE in the placenta after DENV-2 infection (15), suggesting that NE may be transported to the placenta through uterine blood vessels in response to recruitment signals from the uterus, causing placental damage. However, future experiments are required to validate the associations and mechanistic insights indicated by the pathway analysis.

Notably, controversy remains regarding the origins of uNK cells, and this has not yet been fully clarified. Current research offers three main hypotheses (16): (i) progenitor NK cells residing in the uterus before pregnancy (ii), differentiation from CD34^+^ hematopoietic progenitor cells, and (iii) phenotypic changes in pbNK cells. In this study, we observed a high degree of similarity in phenotype between uNK2 cells and pbNK cells. Second, after pregnant mice were subjected to NK cell depletion, the number of uNK cells was reduced compared with that in the DENV-2-infected group and was comparable to that in the uninfected group. This may be due to the complete depletion of pbNK cells. These findings suggest that pbNK cells may convert into uNK cells, supporting the third hypothesis and serving as an important source of the diversity of the uNK cell phenotype.

Although this study provides a new perspective on the mechanism of adverse pregnancy caused by DENV-2, we still need to clarify the potential limitations. First, this study is based on a mouse model rather than on human subjects, and it may not fully capture the complex changes that occur during human pregnancy. Type I interferon (IFN-I) signaling is a critical component of the innate immune response to flaviviruses, including DENV. Wild-type mice are naturally resistant to DENV because their STAT2 protein exhibits inherent resistance to DENV NS5. Unlike in humans, DENV NS5 cannot degrade murine STAT2, allowing the IFN-I pathway to be activated normally (71, 72). This intact signaling pathway effectively restricts viral replication. For these reasons, the *Ifnar1*^−/−^ mouse model is widely accepted in flavivirus research, as it supports viral replication and recapitulates key disease phenotypes—such as pregnancy complications (35, 36, 73). Second, the analysis of uNK cell grouping and function in this study was based on scRNA-seq data and lacks relevant validation. This is because the number of uNK cells in late pregnancy is relatively small, making them difficult to isolate. Currently, no good method has been provided to distinguish between the two phenotypes of uNK cells at the organizational level. A greater number of uNK cells extracted from the uterus of primates might contribute to validation in the future. Furthermore, the functional changes in uNK cells in response to DENV infection during early and mid-pregnancy could enhance our understanding of the underlying mechanisms involved.

**Fig 12:**
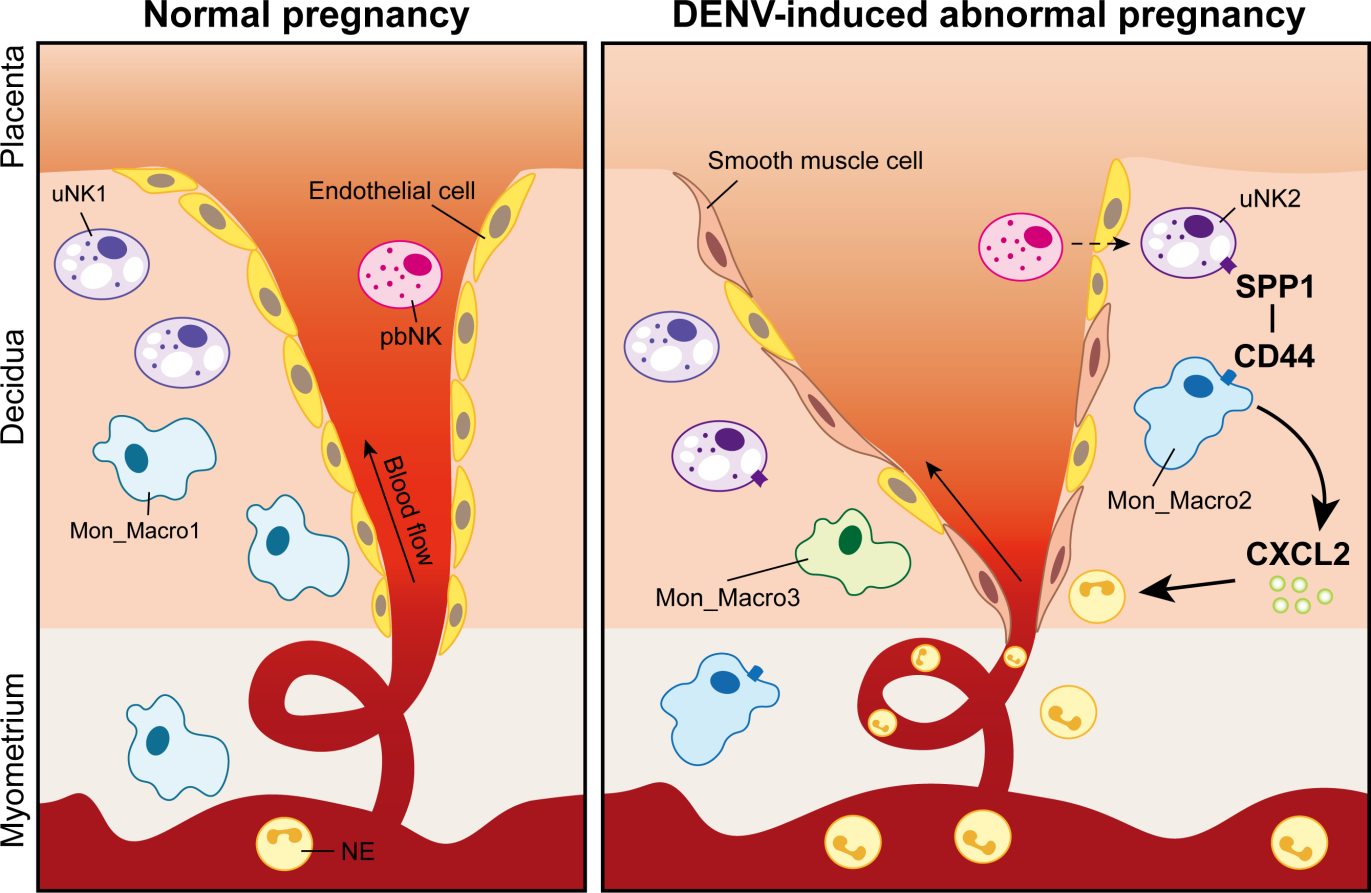
Signaling mechanisms underlying aberrant vascular remodeling in the uterus after DENV-2 infection. Normal pregnancy: Under normal conditions, uterine natural killer (uNK) cells, peripheral blood NK (pbNK) cells, and monocytes/macrophages (Mon_Macro) work in concert to maintain uterine homeostasis and support normal vascular remodeling. DENV-induced abnormal pregnancy: DENV-2 infection triggers a pathological cascade leading to aberrant vascular remodeling and fetal growth restriction. This process is driven by uNK cells—particularly the uNK2 subset—which aberrantly activate monocytes/macrophages (Mon_Macro) via the SPP1–CD44 signaling pathway. Subsequently, these activated Mon_Macro secrete neutrophil (NE)-attracting chemokines, including CXCL2, promoting NE infiltration. This influx of NE ultimately causes uterine tissue damage, impairs vascular remodeling, and results in fetal growth restriction.

In conclusion, this study revealed an association between disrupted uterine immune response-induced abnormal vascular remodeling and fetal growth restriction after DENV-2 infection in an *Ifnar1*^−/−^ mouse model. Notably, the uNK cell subset—uNK2—plays a crucial role in activating inflammatory cascades and promotes the recruitment of NE through interactions with Mon_Macro, potentially serving as a key upstream factor leading to placental vascular damage and adverse pregnancy outcomes (Fig. 12). These findings not only provide reliable evidence for elucidating the pathogenesis of DENV-induced adverse pregnancy but also offer new insights for protecting the health of reproductive women facing DENV infection. Furthermore, these results may have broader implications for understanding and preventing adverse pregnancy outcomes associated with other viral infections.

## MATERIALS AND METHODS

### Dengue virus, cells, and mice

DENV-2 (strain Tr1751) was kindly provided by Dr. Kotaro Yasui previously (74, 75) and has been stored in our laboratory.

The *Aedes albopictus* mosquito cell line (C6/36) was used for DENV-2 propagation. The Vero cell was used to determine viral titers by conducting plaque formation assays.

C57BL/6 mice deficient in interferon α/β receptors (*Ifnar1*^−/−^ mice) were purchased from the Institute of Zoology, Chinese Academy of Sciences. To establish pregnancy, 8- to 10-week-old female *Ifnar1*^−/−^ mice were housed with male *Ifnar1*^−/−^ mice at a ratio of 2:1. The presence of vaginal plugs the following morning was designated as embryonic day 0.5 (E0.5). At E12.5, pregnant mice were infected with 10^5^ plaque-forming units (PFU) of DENV-2 via the footpad injection, with an equal volume (100 µL) of sterile phosphate-buffered saline (PBS) serving as the control. PFU is defined as the number of infectious viral particles that form a plaque in a cell monolayer assay. At E18.5, the mice were euthanized, and maternal blood and critical tissues, including the uterus, were collected for subsequent experiments such as viral load quantification, histological analysis, and RNA sequencing. For the uNK cell depletion experiment, pregnant *Ifnar1*^−/−^ mice from the infection group were randomly selected. Starting from E10.5, Asialo ganglio-N-tetraosylceramide (Asialo-GM1, Thermo Fisher) antibody was administered intraperitoneally once with 30 μg every other day for a total of three injections. The drug was diluted to 100 µL in sterile PBS for administration, while other groups received an equal volume of PBS. The details of the model are shown in Fig. 1.

### Hematoxylin and eosin (HE) staining

Uteruses isolated from DENV-2-infected or uninfected mice were prepared sections with a thickness of 5 μm. Immerse the sections in hematoxylin staining solution to stain the cell nuclei. After staining, decolorize using a 0.5% hydrochloric acid-alcohol solution and rinse with tap water for bluing. Immerse the sections in eosin staining solution to stain the cytoplasm.

### Immunofluorescence (IF) staining and immunohistochemistry (IHC) staining

For IF staining, to visualize the degree of vascular remodeling, the sections were incubated with a rabbit anti-vWF antibody (1:100, Thermo Fisher, PA5-16634), a rabbit anti-α-SMA antibody (1:150, Abcam, ab5694), and a rabbit anti-ZO-1 antibody (1:400, Thermo Fisher, 61-7300) at 4°C overnight. Murine polyclonal antibodies against DENV-2 prepared by the laboratory were used to visualize the distribution of viral antigens in the uterine tissue sections. Then, sections were followed by incubation with a donkey anti-rabbit secondary antibody (1:1,000, Life Technologies, A21207) and mounted with DAPI (ZSGB-BIO, ZLI-9557) for nuclear staining.

For IHC staining, antigen retrieval was conducted using citrate buffer (pH 6.0) or Tris-EDTA antigen retrieval solution (pH 9.0) to re-establish immunoactivity. To assess uterine vasculature damage and immunocyte infiltration after DENV-2 infection, the sections were incubated with a rabbit anti-vWF antibody (1:100, Thermo Fisher, PA5-16634) and a rabbit anti-Ly6g antibody (1:300, Abcam, ab238132) at 4°C overnight. To investigate the activation of the SPP1–CD44 pathway at the histological level, the sections were incubated with a rabbit anti-Osteopontin (SPP1) antibody (1:2000, Abcam, ab283656) and a rabbit anti-CD44 antibody (1:2000, Abcam, ab51037) at 4°C overnight. uNK-positive cells were defined as double-positive for *Dolichos biflorus* agglutinin (DBA) (1:1200, Merck, L6533) and periodic acid schiff (PAS, Servicebio, G1008) staining. The tissue sections were blocked with biotin (Sangon, E674001), incubated with DBA lectin, labeled with horseradish peroxidase (HRP)-streptavidin (1:500, Yeasen, 35105ES60), and subsequently stained with PAS. After that, a secondary HRP-conjugated goat anti-rabbit antibody (ZSGB-BIO, PV-9001) and the chromogen 3,3′-diaminobenzidine (ZSGB-BIO, ZLI-9018) were used to stain the slides. The sections were then stained with hematoxylin to mark the nucleus.

### Remodeling rate calculation

The characteristic of uterine vascular remodeling completion is the loss of vascular smooth muscle cells. Blood vessels showing α-SMA loss were identified as remodeling-finished blood vessels. Remodeling rate = (number of remodeling − finished blood vessels/total number of blood vessels) × 100%.

### Real-time quantitative polymerase chain reaction (RT-qPCR)

Total RNA was extracted using TRIzol (TransGen Biotech, ET101-01-V2), and RNA levels were measured in a 7500 real-time PCR system (Applied Biosystems, USA) using the Quant One Step RT-PCR kit (SYBR Green, TIANGEN, FP313-01). Viral RNA copies were normalized by a standard curve as previously published (15). Viral loads are expressed on a log10 scale as copy number per gram of tissue or per milliliter of blood. The primer sequences targeting *Gapdh*, *MMP3*, *Csf3r*, *Csf3*, *Cxcl12* genes are listed in Table 1.

**TABLE 1:** Primer oligonucleotides used for RT-qPCR

Gene	Primer oligonucleotides
DENV2-F	CATGGGTAACTTATGGGAC
DENV2-R	GTTTCAGTTCGTGTCTCCA
GAPDH-F	GCATTGTGGAAGGGCTCA
GAPDH-R	ACCAGTGGATGCAGGGAT
MMP3-F	GGCCTGGAACAGTCTTGGC
MMP3-R	TGTCCATCGTTCATCATCGTCA
CSF3-F	CATGAAGCTAATGGCCCTGC
CSF3-R	CAGGGACTTAAGCAGGAAGC
CSF3R-F	CAAGTTCACCAGGCAGGTAAG
CSF3R-R	TCGATGTGTCCACAGCTCTC
CXCL12-F	CTGGAGAAAGCTTTAAACAAGGGGC
CXCL12-R	GGCAGGAAGCGGGGAACT

### Transcriptome sequencing (RNA-seq)

Uteruses isolated from pregnant mice were homogenized in TRIzol, and total RNA was extracted according to the manufacturer’s instructions. The purity and concentration of RNA were verified by a NanoPhotometer. The RNA integrity was assessed by electrophoresis (1% agarose gels) using an Agilent 2100 bioanalyzer. mRNA was enriched using Oligo(dT) magnetic beads and served as the template for the synthesis of the first strand of cDNA. The second strand of cDNA was subsequently synthesized using DNA polymerase I with dNTPs as substrates. Double-stranded cDNA was purified and amplified, and only samples that passed quality control were used to construct sequencing libraries. Library construction, quality control, and sequencing were performed by Beijing Novogene Co., Ltd. Differentially expressed genes (DEGs) were screened based on adjusted *P* value (padj) < 0.05 and |log2 fold change| > 0. The DEGs were then subjected to Gene Ontology (GO) enrichment analysis (76).

### Flow cytometry

Fresh uterine tissues were digested using 0.25% trypsin. After incubation with Fc Block (BD, 553142), cells were stained for surface markers using standard dilutions of anti-CD45-APC (eBioscience, 17-0451-82), anti-CD3e-FITC (BD, 553061), and anti-NK1.1-PE (eBioscience, 12-5941-82) antibodies. Finally, the stained cells were read on a DxFLEX flow cytometer (Beckman Coulter, USA) and analyzed using CytExpert software.

### Single-cell RNA sequencing (scRNA-seq)

Uterine tissues were dissociated to prepare single-cell suspensions. Cell viability was required to be >85%, and the cell concentration was maintained between 700 and 1,200 cells/μL. cDNA amplification, library construction, and sequencing were performed according to the manufacturer’s standard protocols (LC-Bio Technology, China). Results from Illumina sequencing offline were converted to FASTQ format using bcl2fastq software (version 5.0.1). The scRNA-seq sequencing data were compared to reference genome using CellRanger software, and cellular and individual cellular 3′ end transcripts were identified and counted in the sequenced samples (https://www.10xgenomics.com/support/software/cell-ranger/latest). The output CellRanger expression profile matrix was loaded into Seurat (version 4.1.0) for filtering of low-quality cells from scRNA-seq data, and the filtered data were downscaled and clustered. Filtering low cell quality thresholds: number of genes expressed per cell >500, mitochondrial genes expressed in <25% of cells. Gene expression values were calculated by the LogNormalize method of Seurat’s “NormalizeData” function. Principal component analysis was performed on the normalized expression values. Uniform Manifold Approximation and Projection (UMAP) was utilized for the visualization of scRNA-seq data. Clustering analysis was performed to identify similar cell subpopulations, which were subsequently annotated based on the expression of marker genes. Expression of upregulated and downregulated genes, analyzed by GO enrichment analysis, was conducted using the clusterProfiler R package v.4.2.256. Intercellular communication in the uterus was analyzed by using CellChat v.1.4.0 as previously reported (77).

### Quantification and statistical analysis

Pathological analyses were performed under the supervision of trained pathologists. Each point in the column graph represents one mouse, with all experiments comprising five biological replicates (*n* = 5). For each mouse, one uterus was processed to prepare three randomly selected paraffin sections, and three fields per section were quantitatively analyzed. The nine measurements were consolidated into a single data point per biological replicate.

To quantify the immunohistochemical results, vascular area measurements were performed using ImageJ software, while the number of uNK cells was manually conducted by at least two independent researchers.

Data visualization and statistical analyses were performed in GraphPad Prism 9.0. Normality of data was tested, and if a nonnormal distribution was found, the nonparametric test was used, such as Mann-Whitney *U*-test for two groups and ordinary one-way ANOVA test for three groups. Data are presented as the mean ± standard deviation (SD). *P* < 0.05 was considered statistically significant.

## Data Availability

The raw RNA data has been uploaded to the China National Microbiology Data Center (NMDC), where individuals and organizations can freely access and download our data. The transcriptome data is available at https://nmdc.cn/resource/attachment/detail/NMDCX0002223. The single-cell RNA sequencing data is available at https://nmdc.cn/resource/attachment/detail/NMDCX0002222. All the other data supporting the findings of this study are available within the article. For additional information or requests, please contact the corresponding author.
